# Whole-genome transcriptomic insights into protective molecular mechanisms in metabolically healthy obese African Americans

**DOI:** 10.1038/s41525-018-0043-x

**Published:** 2018-01-29

**Authors:** Amadou Gaye, Ayo P. Doumatey, Sharon K. Davis, Charles N. Rotimi, Gary H. Gibbons

**Affiliations:** 1 Metabolic, Cardiovascular and Inflammatory Disease Genomics Branch, National Human Genome Research Institute, Bethesda, MD USA; 2 Center for Research on Genomics and Global Health, National Human Genome Research Institute, National Institutes of Health, Bethesda, MD USA; 3 National Heart, Lung, and Blood Institute, National Institutes of Health, Bethesda, MD USA

## Abstract

Several clinical guidelines have been proposed to distinguish metabolically healthy obesity (MHO) from other subgroups of obesity but the molecular mechanisms by which MHO individuals remain metabolically healthy despite having a high fat mass are yet to be elucidated. We conducted the first whole blood transcriptomic study designed to identify specific sets of genes that might shed novel insights into the molecular mechanisms that protect or delay the occurrence of obesity-related co-morbidities in MHO. The study included 29 African-American obese individuals, 8 MHO and 21 metabolically abnormal obese (MAO). Unbiased transcriptome-wide network analysis was carried out to identify molecular modules of co-expressed genes that are collectively associated with MHO. Network analysis identified a group of 23 co-expressed genes, including ribosomal protein genes (RPs), which were significantly downregulated in MHO subjects. The three pathways enriched in the group of co-expressed genes are EIF2 signaling, regulation of eIF4 and p70S6K signaling, and mTOR signaling. The expression of ten of the RPs collectively predicted MHO status with an area under the curve of 0.81. Triglycerides/HDL (TG/HDL) ratio, an index of insulin resistance, was the best predictor of the expression of genes in the MHO group. The higher TG/HDL values observed in the MAO subjects may underlie the activation of endoplasmic reticulum (ER) and related-stress pathways that lead to a chronic inflammatory state. In summary, these findings suggest that controlling ER stress and/or ribosomal stress by downregulating RPs or controlling TG/HDL ratio may represent effective strategies to prevent or delay the occurrence of metabolic disorders in obese individuals.

## Defining the molecular basis of “healthy obesity”

Decreased expression of genes encoding ribosomal proteins could be a hallmark of metabolically healthy obese (MHO) individuals. Amadou Gaye, Ayo P. Doumatey and colleagues at the National Institutes of Health in Bethesda examined the gene expression patterns in whole blood samples from 29 obese African Americans. Eight of the 29 individuals had been categorized as MHO—despite having a Body Mass Index that fell within the obese range they did not have metabolic complications commonly associated with obesity such as diabetes and hypertension. Eighteen ribosomal protein genes were under-expressed in these individuals compared with the 21 that had metabolic disorders. Ribosomal proteins are required for ribosome assembly and protein production and have been implicated in ribosomal stress, which triggers chronic inflammation. These findings reveal a protective mechanism that could prevent or delay obesity-related metabolic comorbidities.

## Introduction

Obesity is a common complex trait with heterogeneous etiologies and manifestations as illustrated by the fact that not all obese individuals develop known metabolic consequences such as insulin resistance, type 2 diabetes and dyslipidemia. This observation has led to the notion that “not all obese humans are created equal”^1^ and the classification of subjects with obesity into at least two major subgroups: the metabolically healthy obese (MHO) and metabolically abnormal obese (MAO). The prevalence of MHO, which is heavily influenced by age and ethnicity, varies widely from a low of about 10% to a high of about 75% across studies.^2^ Recently, Doumatey et al. described this phenotype in a well-characterized population-based cohort of African Americans enrolled from Washington, DC as part of the Howard University Family study (HUFS). The prevalence of MHO was 28% in HUFS^3^ and these subjects displayed paradoxical hyperadiponectinemia (higher than normal adiponectin levels), high HDL-C and normal triglycerides, glucose, and insulin levels.^3^

Although a number of studies have proposed clinical guidelines—similar to those used to define metabolic syndrome^4,5^—to distinguish MHO from other subgroups of obesity, these guidelines do not explain the molecular mechanisms by which MHO individuals remain metabolically healthy despite having a high fat mass. The challenge is to elucidate molecular mechanisms that protect or delay the occurrence of obesity-related co-morbidities in MHO. Previous studies have focused on candidate genes or pathway driven approaches in the attempt to providing insight into the biology of MHO.^6^ In addition, a number of hypotheses have been evaluated in animal models.^7^ Insights gained from these studies include the observation that adiponectin transgenic and leptin deficient ob/ob mice had higher serum adiponectin level and normal insulin sensitivity in the presence of morbid obesity compared to their ob/ob littermate. Additionally, these mice had a much higher proportion of subcutaneous adipose tissue and low systemic inflammation.^5,8^ Interestingly, persons with MHO also display more subcutaneous adipose tissue and less abdominal fat compared to those with MAO.^9,10^ While these studies have provided some insights into molecular mechanisms associated with the MHO phenotype, new opportunities are presented with increasing access to high throughput molecular tools, especially the “omics” as exemplified by the few published mechanistic studies of the MHO phenotype.^11–13^ However, most of these studies were conducted in adipose tissue, an important tissue in metabolic disorders but not easily obtainable. Therefore, it is important to investigate the biological mechanisms underlining MHO using minimally invasive samples such as peripheral blood.

In this study, we investigated the existence of specific set of genes in whole blood with different expression profiles in MHO compared to MAO. We hypothesized that these genes may play a role in protecting individuals with obesity from developing metabolic disorders or in delaying the onset of these disorders. We propose a global and unbiased approach of whole genome mRNA analysis using the most comprehensive MHO definition and leveraging three statistical methods with particular emphasis on network analysis given the interplay of cellular systems and molecular pathways governing metabolism in obese individuals.^14^

## Results

### Weighted gene co-expression network analysis

Weighted Gene Co-expression Network Analysis (WGCNA) was conducted to identify network modules (i.e., clusters of co-expressed genes) that are associated with MHO. Gene ontology (GO) enrichment and Ingenuity Pathway analyses were carried out to determine biological pathways and GO terms enriched in the modules significantly associated with MHO.

#### Network modules correlated with MHO status

Quality control measures (QC) applied to the input data, including graphs and evidence of scale-free topology network for the WGCNA, are described below in the methods section and in the Supplementary Material S1 (Figures S1 and S2). After QC, 29 samples and 14,973 of the initial 27,939 genes were taken forward for further analyses. A total of 36 modules (i.e., clusters of co-expressed genes) were identified after hierarchical clustering and merging of network modules with similar expression profiles (Section 1, Figures S3 and S4, Supplementary Material S1). Correlation coefficients between MHO status and each of the 36 modules in the network were evaluated with p-value and false discovery rate (FDR) provided for each estimate. The relationship between a module and MHO is reported as significant if (1) FDR ≤ 0.05 and (2) the correlation between Module Membership (MM) and Gene Significance (GS) is positive and has a *p*-value ≤ 0.05 (MM and GS are explained in paragraph 3 of WGCNA description in the methods section).

Two modules that included respectively 23 co-expressed genes (lightpink module) and 50 co-expressed genes (khaki4 module) were significantly negatively correlated with MHO status after adjusting for gender and multiple testing. These modules which included 18 ribosomal protein genes were under-expressed in the MHO group (MAO, the reference, is coded as 0 and MHO is coded as 1). MM-GS correlation, a metric to check biological plausibility of the association between module and phenotype (see methods section), is 0.6 (*p*-value = 0.0025) for the lightpink module and 0.53 (*p*-value = 0.000075) for the khaki4 module. The list of genes and their MM and GS values for each of the two modules are provided in Supplementary Table T1.

#### Gene ontology terms and pathways enriched in modules associated with MHO status

A total of 149 gene ontology (GO) terms were significantly enriched in the lightpink module. Notably, the top 10 of the 149 enriched GO terms are involved in mRNA translation processes. No GO term was significantly enriched in the khaki4 module (Supplementary Table T2).

A core analysis conducted in QIAGEN’s Ingenuity® Pathway Analysis (IPA) revealed three pathways enriched in the lightpink module:* EIF2* signaling (*p*-value = 2.08 × 10^−34^), regulation of* eIF4* and *p70S6K* signaling (*p*-value = 7.82 × 10^−11^) and *mTOR* signaling (*p*-value = 4.14 × 10^−10^). We a observed strong evidence of inhibition of *EIF2* signaling (*z*-score = −3.32) pathway in contrast to the *mTOR* signaling and the regulation of *eIF4 and p70S6K* signaling pathways that did not display evidence of activation or inhibition. No known pathway was significantly enriched in the khaki4 module.

In the same IPA analysis, the genes MYCN (*p*-value = 2.94 × 10^−22^) and MYC (*p*-value = 3.29 × 10^−3^) were identified as significantly enriched upstream regulators. MYCN regulates 14 of the ribosomal genes in the lightpink module while MYC regulates 5 ribosomal genes in the same module. MYCN and MYC were more expressed in the MHO group, particularly MYCN (log2 fold change = 0.75). Both differential expressions were not statistically significant but it is known that an upstream transcription factor does not necessarily need to exhibit a large magnitude differential expression to have a major effect on downstream target.

### Differential expression analysis

While the aggregated expressions of specific sets of genes were associated with MHO status in the network module analysis, not all genes in the networks are differentially expressed between MHO and MAO. We therefore evaluated differential expression of each gene in the MHO associated modules. We evaluated the statistical power to detect differential expression (DE) using equal numbers of cases (eight MHO) and controls (eight MAO). The results of that power analysis showed power ≥ 0.76 (FDR < 0.15) is achieved to detect an absolute log fold change ≥ 0.14 for genes with an average expression ≥ 80 read counts (Figure S5 and Table S1, Section 2, Supplementary Material S1). Reassuringly, the differentially expressed genes in the modules associated with MHO status were all in the expression level where power is ≥0.80.

**Fig. 1:**
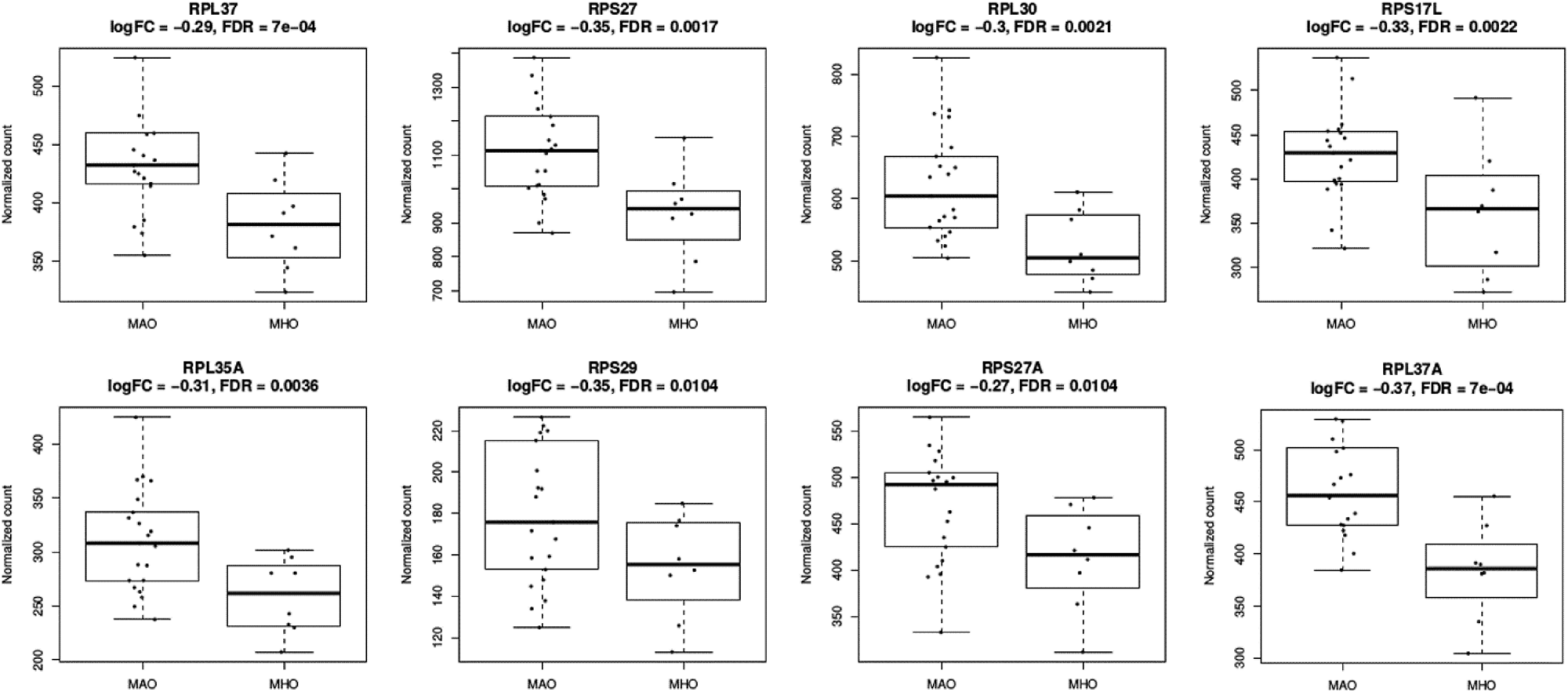
Plots of the normalized expression of the top 7 DE genes in the lightpink module and the top gene (RPL37A) in the khaki4 module

A total of 17 genes out of the 23 in the lightpink module and 32 out of the 50 in the khaki4 module were significantly (FDR ≤ 0.05) differentially under-expressed in the MHO group. Notably, the top 15 genes, by FDR, in lightpink module and the top gene (RPL37A) in the khaki module were all ribosomal protein genes (Fig. 1). The full DE results are reported in Supplementary Table T3.

#### Technical validation of the differential expression analysis

To confirm the expression changes identified by sequencing, we carried out a quantitative RT-PCR of the top eight DE transcripts and one of the two upstream regulators in a subset of subjects (three MHO and nine MAO) for whom RNA samples were still available after RNA-seq. Clinical and anthropometric characteristics of the validation set are provided in Table S2, Section 2, Supplementary Material S1. Overall, the direction and magnitude of the normalized expression fold change (FC) obtained from qRT-PCR were comparable to those obtained by RNA-seq (Figure S6, Section 2, Supplementary Material S1). Additionally, a scatter plot between FC (qRT-PCR) and FC (RNA-seq) displayed a linear relation with all data points falling within the 95% confidence interval (Figure S7, Section 4, Supplementary Material S1).

#### Gene expression and protein levels of ADIPOQ—an anti-inflammatory gene

We analyzed differences in gene expression and circulating protein levels of ADIPOQ between MHO and MAO to validate the working hypothesis that obesity promotes endoplasmic reticulum (ER) stress which promotes decrease in adiponectin mRNA expression as well as decreased multimeric adiponectin. The anti-inflammatory function of the gene is well-documented in the context of obesity and metabolic disorders. This protective function involves the ER stress pathways including EIF2 signaling^15^ that was enriched in our lightpink module. The results showed that MHO subjects under-expressed ribosomal proteins which are key players in the pathophysiology of ER stress. Our linear regression analyses with adjustment for gender revealed higher ADIPOQ gene expression (log fold change = 0.38, *p*-value = 0.02) in MHO compared to the reference group, MAO (Figure S8, Section 2, Supplementary Material S1). The levels of both total (geometric mean of 5419.8 ng/ml vs 5188.1 ng/ml) and high-molecular weight, HMW, (geometric mean of 2921.1 ng/ml vs 2601.1 ng/ml) adiponectin were higher in the MHO group compared to the MAO groups. Consistent with expectation, total and HMW adiponectin protein levels and ADIPOQ gene expression were negatively correlated with TG/HDL (Figure S9, Section 2, Supplementary Material S1).

### Random forest (RF) analysis

We conducted random forest (RF) analyses to evaluate how well genes in the two MHO associated modules can predict MHO. First, we ran RF analyses that included all genes as predictors separately for each module for all 29 subjects. Then, we excluded genes that displayed no predictive power (i.e., variable importance measure, VIM ≤ 0). We subsequently ran variable selection using random forest (VSURF) to identify the smallest subset of genes that achieved the largest area under the curve (AUC; Figure S10, Section 3, Supplementary Material S1). Finally, we were mindful of the fact that our comparison group included unequal number of subjects (MAO > MHO) by applying an RF method that balanced the prediction error.

#### Random forest results for the lightpink module

**Fig. 2:**
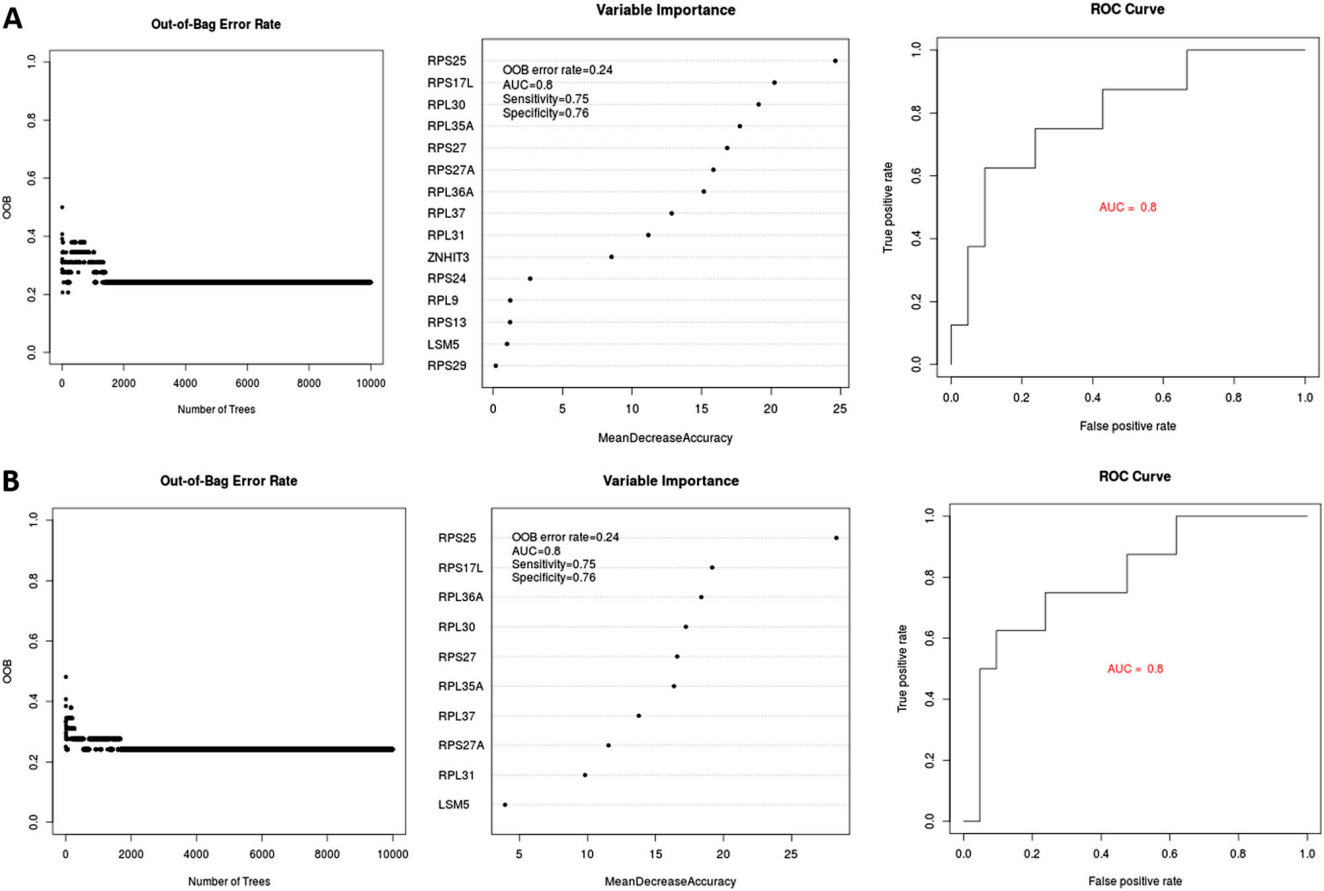
Model performance and ranking of (**a**) 15 genes from the lightpink module and (**b**) a subset of 10 genes identified through variable selection that collectively predict MHO with the same performance

The 23 genes in the module predicted MHO status with AUC = 0.73, out-of-bag (OOB) error = 0.31, sensitivity = 0.62, and specificity = 0.76. A subset of 15 genes with VIM > 0 predicted MHO status with AUC = 0.8, OOB error = 0.24, sensitivity = 0.75, and specificity = 0.76. These results along with the receiver operating curve and the ranking of the 15 genes are displayed in Fig. 2a. Variable selection using VSURF identified a smaller subset of 10 genes that predicted MHO status with model performance values AUC = 0.8, OOB = 0.2, sensitivity = 0.75, and specificity = 0.76. These performance values are identical to values obtained from the analyses that included the larger set of 15 genes (Fig. 2b).

#### Random forest results for the khaki4 module

The 50 genes in this module predicted MHO status with the following performance values: AUC = 0.68, OOB error of 0.28, sensitivity of 0.50, and specificity of 0.81. The analysis of a subset of 27 genes with VIM > 0 resulted in moderately improved prediction values (AUC = 0.72, OOB error = 0.24, sensitivity = 0.50, specificity = 0.86). Furthermore, variable selection using VSURF identified a subset of 19 of the 27 genes, that predicted MHO status with AUC = 0.74, OOB error = 0.24, sensitivity = 0.50, and specificity = 0.86. Compared to the lightpink module, genes in the khaki4 module did not perform as well in predicting MHO status. However, it is important to note that the top predictor (RPL37A) in the khaki4 module was consistent across all 3 RF runs.

#### Evaluation of metabolic parameters driving the observed association between the lightpink module and MHO phenotype

**Fig. 3:**
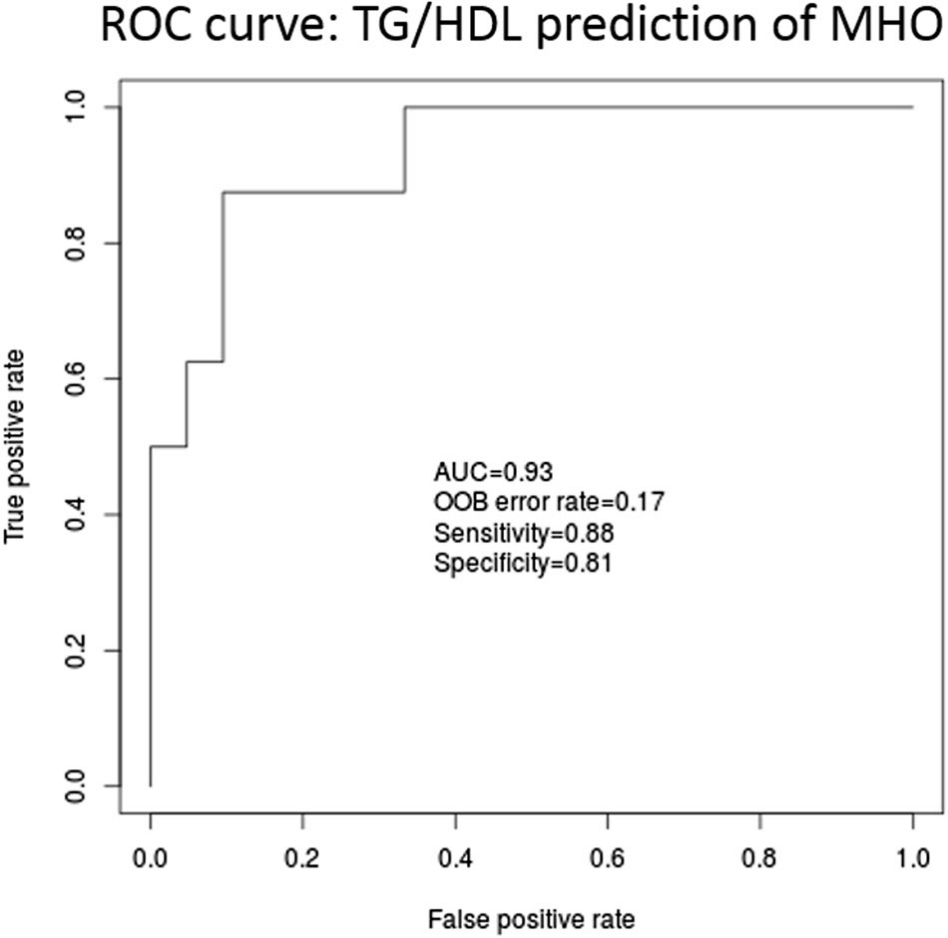
Prediction of gene expression using individual components of MHO definition: TG/HDL ratio is the best predictor of MHO status

Since the definition of MHO is a composite of four parameters including inflammation (CRP), lipids (TG/HDL ratio), fasting glucose, and insulin resistance (HOMA-IR), we conducted nested random forest regression analyses to identify which of these parameters is the primary driver of the expression of the 23 genes in the lightpink modules. The lipid component (TG/HDL ratio) was by far the best predictor of the expression of all but two of the genes in the lightpink module (Table S3, Section 3, Supplementary Material S1). The TG/HDL ratio was also the best predictor of MHO with an AUC of 0.93 (Fig. 3) compared to an AUC of 0.85 for the model that included all the MHO components (Figures S11 and S12, Section 3, Supplementary Material S1).

## Discussion

We analyzed genome-wide transcriptome sequenced data from whole blood to identify networks of co-expressed genes displaying different expression profile between obese individuals without metabolic complications (MHO) and MAO individuals. We discovered two network modules one with 23 genes (lightpink module) and the other with 50 genes (khaki4 module) that were significantly downregulated in MHO subjects. GO analysis revealed an enrichment of terms related to mRNA translation in the lightpink module where 18 of the 23 genes are ribosomal protein genes (RPs). Three pathways were significantly enriched in the lightpink module, *EIF2* signaling, regulation of *eIF4* and* p70S6K* signaling, and *mTOR* signaling. Two genes, MYCN and MYC, were identified as upstream regulators of 14 and 5 RPs genes respectively in the lightpink module. Furthermore, differential expression analyses of each gene revealed that 17 (16 RPs) of the 23 genes within the lightpink module and 32 of the 50 genes in the khaki4 module were significantly differentially expressed between MHO and MAO subjects.

Given that the signals with MHO were statistically stronger for the lightpink module, we focused further discussion on these findings. The TG/HDL ratio, an index of insulin resistance, was the best predictor of the expression of genes in this module and seems to be the primary driver of the relationship between this module and the MHO phenotype. Moreover, a subset of 10 co-expressed RPs genes in this module was highly accurate in predicting MHO status with AUC = 0.8. These findings provide plausible biological mechanisms for the MHO phenotype.

RPs are key players in the translational machinery of mammalian cells and participate in ribosomal biogenesis. They are biologically active during cell growth, proliferation, and development.^16^ While evidence is available for their role in disease etiology including cancer, their role in human metabolic diseases is not well established. However, some insights have been gained from mouse model with the homozygous RPL29-knockout mice displaying low body weight at birth and global skeletal growth defects.^16,17^

The involvement of RPs in immune signaling especially in the context of inflammation in innate immune response is particularly relevant in the context of the present investigation. Obesity is characterized by a dynamic adipose tissue remodeling including hyperplasia (increased number of adipocyte), hypertrophy (enlargement of adipocytes) and chronic low-grade inflammation.^18^ The increase in cell mass that occurs during extensive cell proliferation, such as adipocyte hyperplasia and hypertrophy, requires increased protein synthesis, a process that depends on a steady supply of ribosomes; hence the coupling of ribosome biogenesis and protein synthesis in cell cycle.^19,20^ Our results indicate an upregulation of several RP genes among the subjects with metabolic disorders (MAO) suggesting a mechanism developed by the body to cope with the increased cellular and metabolic stresses associated with obesity. Increased protein synthesis and activities have shown to result in rapid accumulation of misfolded proteins that can trigger ER and ribosomal stresses^16,21,22^ with detrimental effects to the cell or organism.^23^ Ribosomal and ER stress can trigger inflammation, a natural process, through which the body attempts to remove pathogens and cell debris. However, ineffective control of these processes can lead to chronic inflammatory state with serious metabolic consequences.^23^

Our observation of a higher expression of RPs and dysregulation of ER-associated canonical pathways (i.e., EIF2 signaling, mTOR signaling, regulation of eIF4 and p70S6K signaling) indicate increased ribosomal and ER stress and higher inflammation in the unhealthy obese group (MAO). In contrast, we observed lower expression of RPs, lower inflammation and higher anti-inflammatory status as measured by higher ADIPOQ expression and serum adiponectin levels among the healthy obese subjects. These observations suggest that MHO subjects may be more physiologically effective in modulating both ER and ribosomal stress with an attenuation of the inflammatory state characteristic of MAO individuals. This observation is consistent with the results of a study conducted in Saccharomyces cerevisiae which showed that reduced translation or deficiency in ribosomes protects against ER stress.^24^ Furthermore, it has been reported that ER stress leads to reduced adiponectin secretion with resulting increased inflammation in human adipocytes.^15^

We note that TG/HDL ratio appears to be a major driver of the change in the expression profile of the RPs between MHO and MAO subjects. Hence, we postulate that increased TG/HDL ratio, an indicator of insulin resistance, likely represents a deterministic stimulus in the triggering of ER and ribosomal stresses with inflammatory consequences in obesity. Indeed, the TG/HDL ratio is 2.3 times lower in MHO than in MAO. Thus, our findings not only support the role of inflammation in the development of metabolic disorders in obesity, but also provide novel insight into the etiologic pathways that link obesity, perturbations in cellular metabolism, and chronic inflammation.

Upstream analysis identified conserved transcription factors (TFs), MYCN and MYC as regulators of RPs. These TFs are known to modulate cell proliferation and regulate ribosome biogenesis and protein synthesis,^25^ and there is evidence now they may be involved in metabolic reprogramming through lipid metabolism.^26^ Down-regulation of RPs seems to be a homeostatic protective mechanism that appears to be intact in MHO; but perturbed in MAO such that there is chronic inflammation with its MAO-related consequences.

This study has several strengths including major efforts to avoid the potential of false positive signals by implementing different statistical methods/techniques (WGCNA, RF, and DE), and stringent QC measures. This robust and well-integrated analytical framework represents the first such attempt to identify plausible pathways related to MHO. Although analysis of whole blood provides a good overview of physiologic activities in many tissues, it is however important to note that signals from some tissues may not or may only be partially captured from peripheral blood. Nevertheless, whole blood is a reasonable tissue for the investigation of complex conditions such as metabolic disorders that involve multiple tissues, pathways and cell types. Finally, we recognize that this cross-sectional study cannot infer causality, thus functional assays as well as replication in other populations as more “omics data” become available are warranty.

## Conclusion

The molecular biology technique (RNA-sequencing) implemented in this study to shed novel insights into the molecular basis of MHO in African Americans identified a set of RPs which predicted MHO status with high accuracy. Our findings support previously identified role of inflammation in the development of obesity-related co-morbidities. Notably, we provided novel insights into potential mechanisms by which inflammation is triggered in obesity. Furthermore, we provided evidence in support of the role of ER and ribosomal stresses via RPs in the onset of inflammation in the obesity state. The high TG/HDL-C ratio seen in MAO may suggest activation of ER and related-stress pathways that ultimately lead to chronic inflammatory state. Controlling ER stress and/or ribosomal stress by downregulating RPs with chemical agent(s) or keeping TG/HDL ratio in a “normal range” may represent effective strategies to prevent or treat metabolic consequences in obese individuals. Finally, the set of RPs identified in this study may represent an objective classifier of MHO status following validation in independent studies.

## Materials and methods

### Cohort description

The Minority Health Genomics and Translational Research Bio-Repository Database (MH-GRID) project is a study of severe hypertension in African Americans aged 30–55 years. The data included in this analysis consist of whole blood RNA from a subset of the MH-GRID cohort. Details of inclusion and exclusion criteria for the MH-GRID study are provided in Section 4 of Supplementary Material S1. All participants signed a written informed consent before their participation in the study. The study was approved by the Morehouse School of Medicine, Kaiser Permanente, Grady Health System Research Oversight Committee, and the National Institutes of Health Institutional Review Boards.

MHO subjects were defined based on the third and most comprehensive definition outlined in Table 1. This definition adds inflammatory status to the modified definition by Wildman et al.^27^ MAO subjects were defined as obese (BMI ≥ 30) individuals not meeting the MHO definition. The baseline characteristics of the 29 subjects, matched for age, included in our analysis are reported in Table 2.

**Table 1:** Definitions commonly used in the literature for MHO

Definition 1	Definition 2	Definition 3
Basic MHO definition	Modified Wildman et al. definition	Includes inflammatory status
∙ No hypertension (BP ≤ 130/85 mmHg, no BP medication)	∙ No hypertension (BP ≤ 130/85 mmHg, no BP medication)	∙ Definition 2+
∙ No diabetes (glucose ≤ 126 mg/dl)	∙ No pre-diabetes or diabetes (glucose ≤ 100 mg/dl)	∙ hsCRP ≤ 0.3 mg/dl)
∙ HDL-C ≥ 40 mg/dl for male	∙ HOMA ≤ 5.1	∙ Karelis et al. (cut off for CRP)
HDL-C ≥ 50 mg/dl for female	∙ TG/HDL ≤ 1.65 for male	∙ All conditions must be met
∙ All conditions must be met	TG/HDL ≤ 1.32 for female	
	∙ All conditions must be met

**Table 2:** Baseline characteristics of the phenotype data by MHO and MAO status

Characteristics	Metabolically healthy and obese (MHO)	Metabolically abnormal and obese (MAO)
*N*	8	21
BMI	34 ± 6 (30, 47)*	37 ± 5 (30, 45)
Glucose (mg/dl)	87 ± 6.57 (77, 98)	92.62 ± 9.59 (78, 121)
CRP (mg/dl)	0.13 ± 0.09 (0.04,0.3)	0.36 ± 0.21 (0.07, 0.72)
HOMA-IR	1.99 ± 1.49 (0.3, 4.56)	4.21 ± 2.97 (0.94, 13.12)
TG/HDL ratio	0.98 ± 0.27 (0.52, 1.3)	2.25 ± 1.21 (0.88, 5.14)
Hypertension (Control/case)	8/0	8/13
HMW adiponectin (ng/ml)	3565 ± 2497.5 (1120,8080) [2921.1**]	3453.5 ± 2733.2 (808.82, 10200) [2601.1**]
Total adiponectin (ng/ml)	5878.7 ± 2615.48 (2920,10900) [5419.8**]	5491.4 ± 2042.6 (2960,10600) [5188.1**]
Age (years)	41.88 ± 6.08 (34, 54)	42.33 ± 5.97 (34, 54)
Gender (Female/Male)	3/5	14/7
Current smoker (No/Yes)	4/4	11/10
Regular alcohol drinker (No/Yes)	5/3	15/5

*The minimum and maximum values are between brackets

**Geometric mean

### RNA sequencing data

RNA extraction: Total RNA extraction was carried out using MagMAX^TM^ for Stabilized Blood Tubes RNA Isolation Kit as recommended by vendor (Life Technologies, Carlsbad, CA).

Library preparation: Total RNA samples were converted into indexed cDNA sequencing libraries using Illumina’s TruSeq sample kits. After PCR amplification, the final libraries were quantitated by qPCR (KAPA Library Quant Kit, KAPA Biosystems).

Sequencing strategy: Illumina paired-end 100 base pair sequencing was performed on HiSeq 2000 analyzer (Illumina, USA) with a sequencing depth of 75 million reads per sample. Twelve samples were pooled in equimolar ratios; the quality control of the pooled samples and determination of the loading concentration were performed on MiSeq (Illumina, USA).

Expression quantification: The quantification of mRNA expression was done in three steps; (i) adapter trimming was conducting with *FastqMcf*^28^ to remove remnants of sequencing primers/adapters and low-quality regions from the raw RNA-Seq read data, and improve subsequent alignment rates; (ii) reads were aligned to the transcriptome, using *BowTie2*^29^ and the relevant reference genome (hg38); (iii) finally, the expression levels were measured using the RNA-Seq by expectation maximization method.^30^ The mRNA sequencing data of 29 samples across 27,939 transcripts were analyzed. More details about RNA extraction, library preparation and expression quantification are available in Section 5 of the Supplementary Material S1.

### Quality controls

The expression data were normalized using the weighted trimmed mean of M-values method,^31^ an optimal method for the normalization of mRNA sequencing data. Transcripts that did not achieve 1 count per million (CPM = count/sum [counts] x 1million) in at least three samples were excluded to remove genes with very low expression that are likely to be noise. Principal component analysis was conducted to identify sample outliers.

### Protein measurement

CRP, total and HMW adiponectin were measured per manufacturer’s specifications using magnetic bead-based multiplex assays from R&D systems (Minneapolis, MN) on a Luminex IS100 instrument (Luminex Corp. Austin, TX). The analytes were grouped into panels by the manufacturer based on their abundance in “normal” human serum. The data were analyzed using Bio-Plex Manager Pro 6.1 analysis software (Bio-Rad, Hercules, CA).

### Statistical analyses

**Fig. 4:**
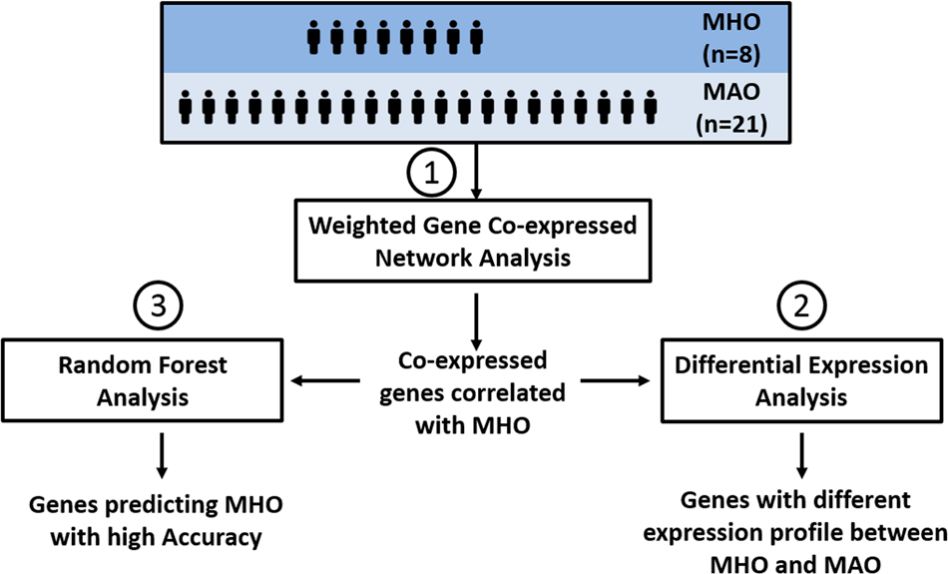
Graphical depiction of the three complementary analytical strategies implemented in this study

Three complementary statistical approaches were used to define the most robust molecular signature of metabolically healthy obesity. The chart in Fig. 4 shows the series of analyses conducted and the methods used to achieve the most reliable results. The three methods are described below.

#### Weighted gene Co-expression Network Analysis (WGCNA)

The aim of this analysis is to investigate the interplay between genes to identify modules (clusters of genes whose expressions are highly correlated) and the relationship between those modules and MHO phenotype. This analysis was conducted following the WGCNA methodology, in the R environment and in three steps detailed elsewhere.^32^ Because the network analysis is essential to this project, the WGCNA analysis steps are described in more details below.

1. Network construction: The network consists of all the genes that passed quality control (QC) filters. In the network, each gene is a node and closely related genes (i.e., co-expressed genes) form a module. First, a co-expression similarity matrix, **s**_**ij**_, that holds correlation values between genes is computed. Then **s**_**ij**_, is transformed into an adjacency matrix, **a**_**ij**_, a matrix that tells whether any two genes have a correlation ≥ *τ*, a threshold to determine if two genes are connected (close). In un-weighted networks **a**_**ij**_ takes the values 0 or 1, as shown in the mathematical expression below, and *τ* is then called a “hard” threshold.
  $${\mathrm{if}}\,{\boldsymbol{s}}_{{\boldsymbol{ij}}} \ge \tau \to {\boldsymbol{a}}_{{\boldsymbol{ij}}} = 1\,{\mathrm{else}}\,{\boldsymbol{a}}_{{\boldsymbol{ij}}} = 0$$
  However, such hard threshold does not reflect the continuous nature of biology. Therefore, weighted networks which allow for **a**_**ij**_ to take any values between 0 and 1 represent a more appropriate framework; *τ* is then called a ‘soft’ threshold. A critical decision is the choice of an appropriate *τ*. An adequate threshold *τ* is one that leads to a scale-free topology network (i.e., a structured network with central (hub genes) and peripheral nodes as opposed to a random network). A characteristic of a scale-free topology network is that the probability that a node has *k* connections follows a power law *P(k)~k*^*-γ*^. For a perfect scale-free topology network the correlation between *P(k)* and *k* is 1. In this analysis, the choice of the threshold was verified to ensure the requirement of a scale-free topology network is fulfilled: *ρP* (*k*), *k* ≥ 0.8.
2. Identification of network modules: After choosing an appropriate threshold, modules were identified through hierarchical clustering using the Unweighted Pair Group Method with Arithmetic Averaging method^33^ to build a dendrogram, a diagram that hierarchically nests genes into increasingly more inclusive clusters. The clustering is based on information from a topology overlap matrix, a matrix that combines co-expression information from the adjacency matrix and topological similarity.^34,35^ The minimum size of the modules was set to 10 to ensure that small as well as large modules are detected. Subsequently, modules with very similar expression (those very close in the dendrogram) were merged.
3. The relationship/association between the modules identified in the previous step and MHO were then investigated. The aggregated expression of each module, the first principal component of the reduced data, termed module eigengene, is computed and its correlation with MHO status determined. The relationship between a module and MHO is reported as significant if 2 conditions are fulfilled:

- The false discovery rate (FDR) adjusted p-value of the correlation is ≤0.05.
- The correlation between Module Membership (MM) and Gene Significance (GS) is >0 with a *p*-value ≤ 0.05. MM is the correlation between the expression profile of a gene and the module eigengene (aggregated expression of all genes in a module, the 1^st^ principal component); MM takes values between 0 and 1 and tells “how well a gene belongs to a module”; hub genes have an MM value closer to 1. GS is the absolute value of the correlation between a gene and the outcome, MHO. The correlation between a module and MHO status is in fact a correlation between the module eigengene and MHO status. Therefore, in a biologically plausible module-phenotype association, hub genes would be more correlated with MHO than genes at the fringes of the module and this leads to a positive correlation between MM and GS.

After identifying modules associated with the outcome of interest, GO enrichment analysis was performed, using the R library limma, to identify GO terms over-represented in each of the modules. The function used computes one-sided hypergeometric tests equivalent to Fisher’s exact test.^36^ The modules were further investigated for the presence of known pathways and upstream regulators, in QIAGEN’s Ingenuity® Pathway Analysis (IPA®, QIAGEN Redwood City, www.qiagen.com/ingenuity).

#### Differential expression analysis

The R library *edgeR* (24) was used to examine differential expression between MHO and MAO. *EdgeR* fits a negative binomial model to transcripts read counts (i.e., expression) and computes likelihood ratio tests for the coefficients in the model. The DE analysis included genes in modules identified as associated with MHO phenotype in the WGCNA analysis. Genes with an absolute log fold change > 0 and FDR ≤ 0.05 are reported as differentially expressed. A deviance of goodness of fit test was run to identify genes with poor model fit indicating that the dispersion estimate of the gene was markedly far from the common dispersion. Dispersion outliers were carefully inspected, because outlying dispersion can indicate either low quality or large expression difference due to artefactual effects.

Power analysis was conducted using the R library PROPER^37,38^ to estimate the power to detect the lowest significant log fold change in the list of differentially expressed genes, with 8 MHO and 8 MAO samples.

#### Differential expression analysis validation

Technical validation of the DE results was carried out with quantitative real-time polymerase chain reaction (qPCR) using three MHO and nine MAO samples, a subset of the initial samples for which RNA was still available. A summary of the characteristics of these 12 samples is available in Section 2 of Supplementary Material S1 (Table S2). Eight of the top differentially expressed genes, based on adjusted p-value, were assayed in addition to the upstream regulator MYCN.

RNA samples were reverse transcribed using Invitrogen SuperScript IV RT cDNA synthesis kit with random hexamer primers following the manufacturer’s instructions (ThermoFisher Scientific, Waltham, MA). The qPCR assay was then carried out on Bio-Rad CFX96 system (BIO-RAD, Hercules, CA) using previously synthesized cDNA and TaqMan gene expression assays which include two unlabeled PCR primers and one FAM® dye-labeled TaqMan® MGB probe (ThermoFisher Scientific, Waltham, MA). A PCR reaction of 2 μl was used for all assays and contained 10 μl of TaqMan® Fast Advanced Master Mix (2×), 1 μl of TaqMan gene expression assay mix (20×) and 9 μl of cDNA diluted in RNase-free water. All samples including the controls (no template controls and reverse transcriptase controls) were run in triplicates. The thermal cycling conditions were as follow: 50 °C for 2 min, 95 °C for 20 s, and [95 °C for 3 s, and 60 °C for 30 s] × 40 cycles.

The qPCR data was analyzed using Relative Expression Software Tool (REST 2009), a stand-alone software developed by Pfaff and Qiagen (http://www.REST.de.com) and uses the ΔΔCt method. The expression values were normalized to two reference genes, *GAPDH* and *ACTB* (ThermoFisher Scientific, Waltham, MA). Transcripts were considered validated if the direction and magnitude of the normalized expression ratios (FC) are consistent between the methods (i.e., RNA-seq and q-PCR).

#### RF analysis

RF is a machine learning technique that makes no assumptions about the relationship (e.g., linear) between the predictors and the outcome and can capture interactions that cannot be easily included in regression models. Genes in each module associated with MHO status were used as predictor variables in a random forest analysis to assess how well they collectively predict MHO. It is reasonable to expect modules or subset of modules correlated with the MHO phenotype to predict MHO status with a high accuracy. For instance, some genes in the modules would each have some predictive power to predict MHO and they collectively could provide a reasonably good classification of MHO. For this analysis the R library *randomForest*, an R implementation of the algorithm developed by Breiman and Cutler,^39^ was used. In RF, cross-validation is not necessary; technically speaking there are no training and test datasets: for each tree, a subset of all the samples, is drawn by sampling with replacement (bootstrap) and the rest of the data are left out; a large forest of 10,000 trees (*ntree*) was generated for robust prediction estimates. For each tree, the number of predictor variables sampled (*mtry*) as candidates at each split is $\frac{p}{2}$ where p is the total number of genes. The samples left out represent the out-of-bag (OOB) set used to get an unbiased estimate of the misclassification error of the tree.^39^

RF provides a Variable Importance Measure (VIM), a score that denotes the variable’s predictive power. VIM is obtained as follows: for each tree, the misclassification rate (error rate) in the OOB set is evaluated (errOOB_1_); then the values of the variable are permuted and after classification, the error rate for that perturbed OOB set (errOOB_2_) is computed. Finally, the error rate in the perturbed set, is subtracted from that of the un-perturbed OOB set; this operation is carried out across all the trees. The VIM formula can be written mathematically as described below where VIM_*k*_ is the raw VIM score of a variable *k*, *ntree* is the number of trees and $errOOB_2^k$, the misclassification error on the perturbed OOB set when the values of the variable *k* are permuted. In our analysis, the number of permutations (*nPerm*) was set to 1000.

$$VIM_k = \frac{{{\sum} {errOOB_2^k} - errOOB_1}}{{ntree}}$$

Although RF ranks the predictor variables (here genes) by VIM, not all the variables with some predictive power (VIM > 0) are truly important; some are noise. Since this project focuses on the most robust molecular signatures, we subsequently searched for the true predictors, a process called variable selection. Variable selection was carried using a method by Genuer *et al*. implemented in the R programming language and described elsewhere.^28,40^ This method has the particularity of using a heuristic approach where the threshold to sift out noise predictors is derived from the data and is hence not an arbitrary cut-off independent of the data.^28^

#### Protocol approval

The study was performed in accordance with relevant guidelines and regulations and approved by the Morehouse School of Medicine, Kaiser Permanente, Grady Health System Research Oversight Committee, and the National Institutes of Health Institutional Review Boards.

### Participants anonymity

Patient identifiers have been removed within the text, tables, figures, and images. All reasonable measures have been taken to protect patient anonymity.

### Data availability statements

The underlying data set necessary for replication of this study is available within the paper and its Supporting Information files (Supplementary_Table_T4.zip).

## Electronic supplementary material

Supplemental Material S1

Supplemental Material T1

Supplemental Material T2

Supplemental Material T3

Dataset 1
