# Supplementary material for: Whole-genome transcriptomic insights into protective molecular mechanisms in metabolically healthy obese African Americans
Source: NPJ Genom Med. 2018 Jan 29;3:4. doi: 10.1038/s41525-018-0043-x (PMC5789085; doi:10.1038/s41525-018-0043-x)
Supplement: Supplementary file 1 — Supplemental Material S1 [file 41525_2018_43_MOESM1_ESM.docx]

Supplementary Material S1

# QUALITY CONTROLS

Transcripts with expression < 1 count per million (CPM =count/sum [counts] x 1million) in at least 3 samples were excluded to keep only genes with expression difference that can be reliably assessed. Figure S1 shows the distribution of read counts before and after filtering of genes with very low expression.

Figure S1: Distribution of read counts before (A) and after (B) filtering out genes with low expression.


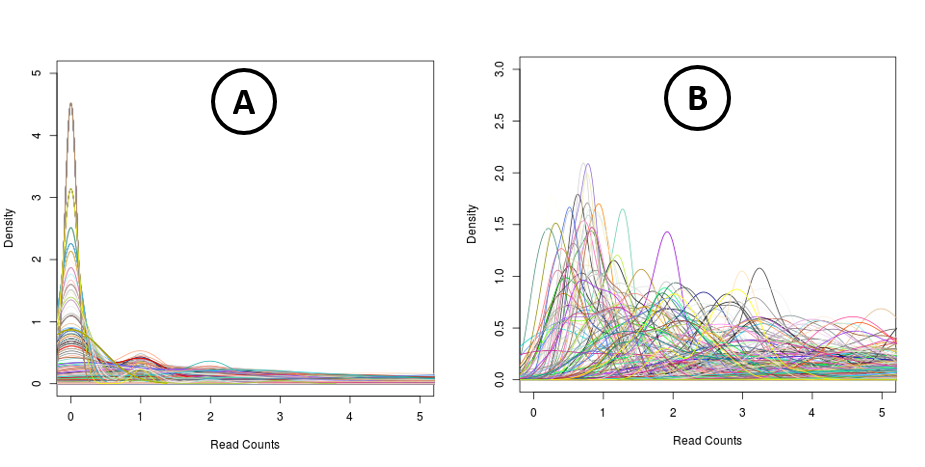


An appropriate soft-threshold that ensures the network of genes has the characteristics of a scale-free network topology was chosen. Figure S2 shows that the relationship between log10(k) and log10(P(k)) follows the power law with a correlation of 0.81 and a soft-threshold of 23.

Figure S2: Plot k vs. P(k) to verify scale-free topology network requirement is fulfilled.


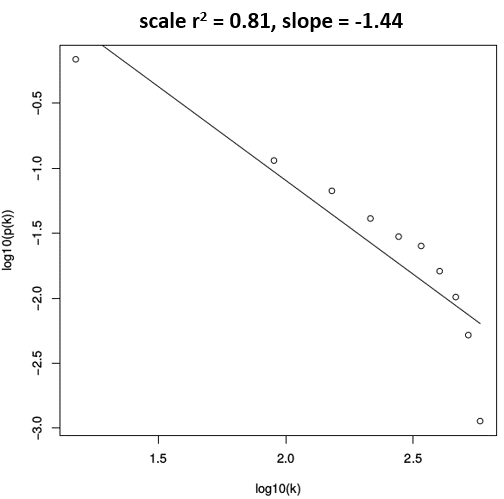


After clustering the 14973 genes that passed QC, 224 modules were identified. Modules with similar expression profiles (module eigengene dendrogram cut-height ≥ 0.8) were subsequently merged as shown in Figure S3 and the resulting 36 modules (Figure S4) were investigated for relationship with the phenotype of interest, Metabolically Healthy Obesity (MHO).

Figure S3: Identification of 224 modules after agglomerative clustering using the complete-linkage method and adaptive branch pruning. The red line indicates the threshold used to merge similar modules based on their expression profiles.


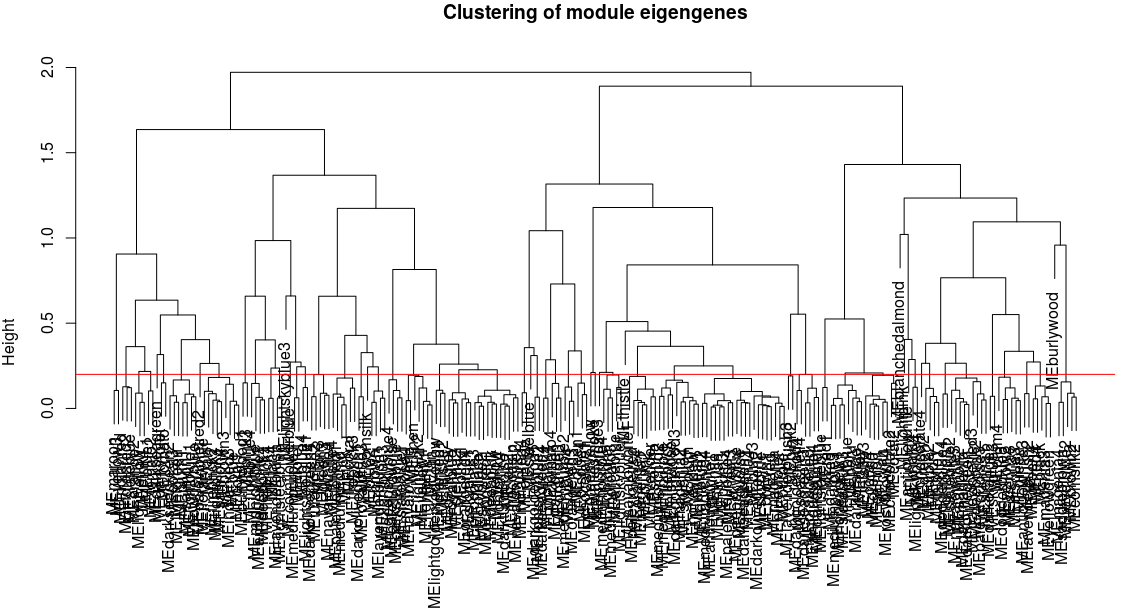


**Figure S4: Identified network modules; the two highlighted modules were correlated with MHO.**


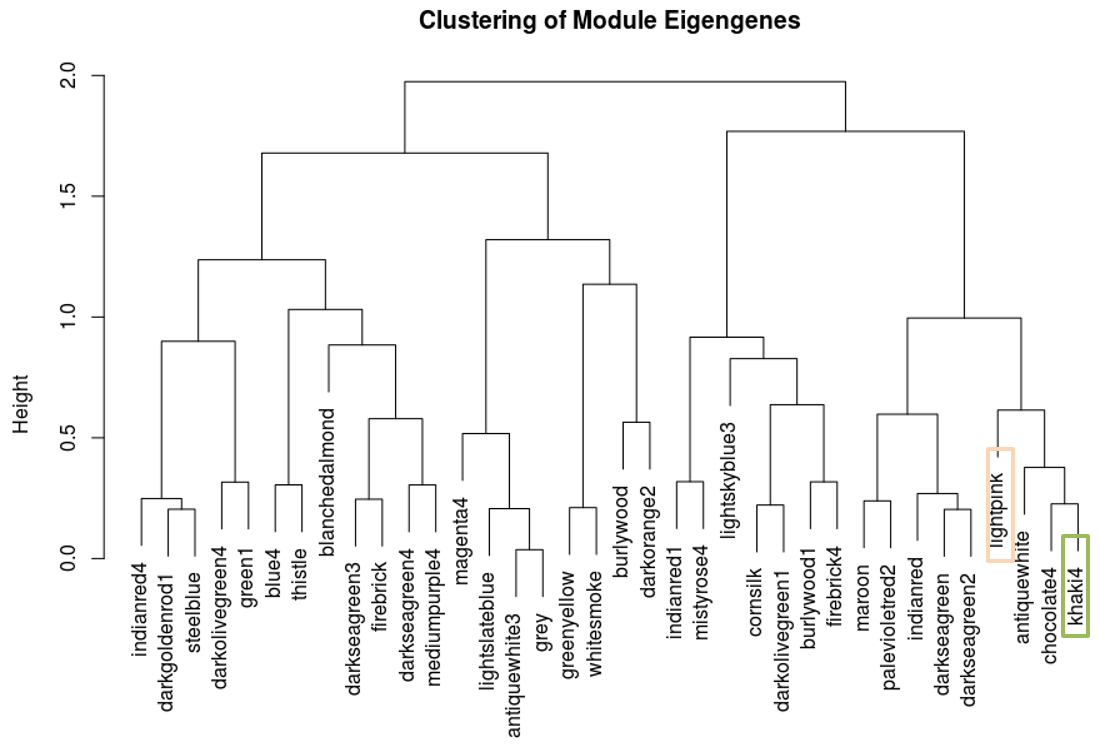


# DIFFERENTIAL EXPRESSION ANALYSIS

**STATISTICAL POWER**

Power analysis was carried out using the R library PROPER. The tool evaluates power empirically and takes into account factors that influence power such as sequencing depth, distribution of the mean expression level, and filtering threshold. Power was assessed by evaluating success rate to detect an absolute log fold change of 0.14, over 500 simulation runs. This absolute log fold change of 0.14 is the lowest fold change among the genes differentially expressed in one of the network modules associated with MHO. Success rate was determined by comparing the p-values to the specified type I error rate (alpha = 0.01) to reject the null hypothesis. In Figure S5, the plots indicate that with 8 MHO and 8 MAO subjects, statistical power ≥ 0.76 and FDR < 0.15 are achieved to identify differentially expressed gene with an average expression ≥ 80 read counts. All the genes in the lightpink module are in the strata with power ≥ 0.80 (Table S1).

Figure S5: Statistical power and false discovery rate (FDR) for several expression strata given the characteristics of the MH-GRID mRNA data.


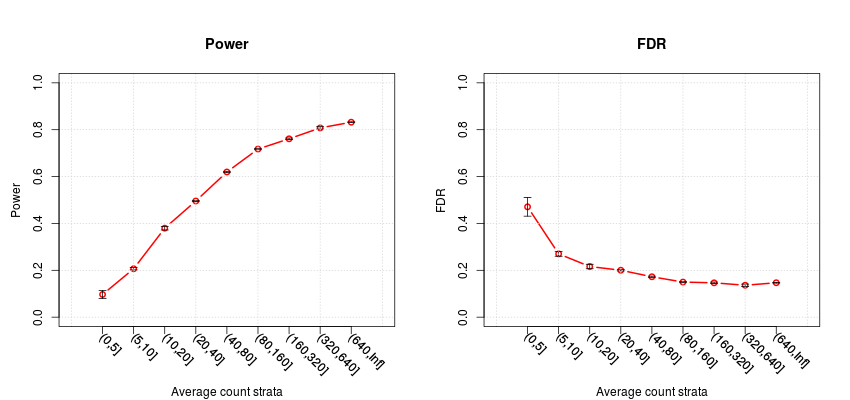


Table S1: Expression levels of genes in the lightpink and khaki4 modules (lightpink module genes are highlighted in grey).

| **Expression strata** | **Genes in the lightpink and khaki4 modules** |
| --- | --- |
| (0, 5] | - |
| (5, 10] | - |
| (10, 20] | - |
| (20, 40] | - |
| (40, 80] | - |
| (80, 160] | BRMS1L, C11orf1, ZNF280B |
| (160, 320] | PPIH, ZNF382, TUBE1, ZNF470, GORAB |
| (320, 640] | CHCHD7, ZNF573, DNAJC24, ZNF675, MRPS31, MRPL45, HOOK1, C5orf28, ZNF709, ERCC8, **LSM5**, C12orf47, ZNF383, TSEN15, SNHG8, C4orf43, PEX3, C14orf126, IQCB1, **ZNHIT3** |
| (640, Inf] | RPL37A, **RPL37**, **RPS27**, **RPL30**, **RPS17L**, **RPL35A**, CASD1, RPL7, RBMX, **RPS27A**, **RPS29**, SYNJ2BP, ELK4, **RPS25**, **RPL24**, **RPL34**, **RPL31**, **RPL36A**, LYRM7, RMP, **RPL26**, FASTKD2, DPY19L4, ZNF260, DNAJC16, NACA, AGL, ZNF681, KLHL20, **RPL9**, **RPS13**, **COMMD6**, RABL3, MEAF6, **RPL38**, ANKRD40, **SNHG6**, ZNF480, **RPL41**, CALCOCO1, NARG2, TSC22D3, ZBTB10, RABGGTB, **RPS24** |

**VALIDATION OF SELECTED DIFFERENTIALLY EXPRESSED GENES by RT-PCR**

Table S2: Baseline characteristics of the 12 samples included in the validation of the differential expression analysis.

| **Characteristics** | **Metabolically Healthy and Obese (MHO)** | **Metabolically Abnormal and Obese (MAO)** |
| --- | --- | --- |
| N | 3 | 9 |
| BMI | 37 ± 9 (30, 47)* | 37 ± 4 (32, 44) |
| Glucose (mg/dl) | 88.33 ± 10.6 (77, 98) | 95.78 ± 12.15 (82, 121) |
| CRP (mg/dl) | 0.08 ± 0 (0.07, 0.08) | 0.42 ± 0.24 (0.07, 0.72) |
| HOMA-IR | 2.87 ± 2.26 (0.3, 4.56) | 4.76 ± 3.73 (0.94,13.12) |
| TG/HDL ratio | 0.88 ± 0.31 (0.52, 1.09) | 2.48 ± 1.56 (0.88, 5.14) |
| Hypertension (Control/Case) | 3/0 | 3/6 |
| HMW Adiponectin (ng/ml) | 5306.7 ± 3689 (1120, 8080) [3932.5**] | 3768.2 ± 33.30 (863.8, 10200) [2660.6**] |
| Total Adiponectin (ng/ml) | 7656.7 ± 3552.5 (3860, 10900) [7016.5**] | 6107.8 ± 2314.8 (3690, 10600) [5754.7**] |
| Age (years) | 41.67 ± 3.51 (38, 45) | 42.11 ± 2.8 (37, 45) |
| Gender (Female/Male) | 1/2 | 5/4 |
| Current smoker (No/Yes) | 2/1 | 6/3 |
| Regular alcohol drinker (No/Yes) | 2/1 | 6/2 |
| * The minimum and maximum values are between brackets. ** Geometric mean. | | |

Figure S6: Relative expression of top 8 differentially expressed transcripts (DET) plus expression of predicted upstream regulator of DET (MYCN) in whole blood of MHO versus MAO determined by RT-PCR (blue bar) and RNA-seq (red bars).


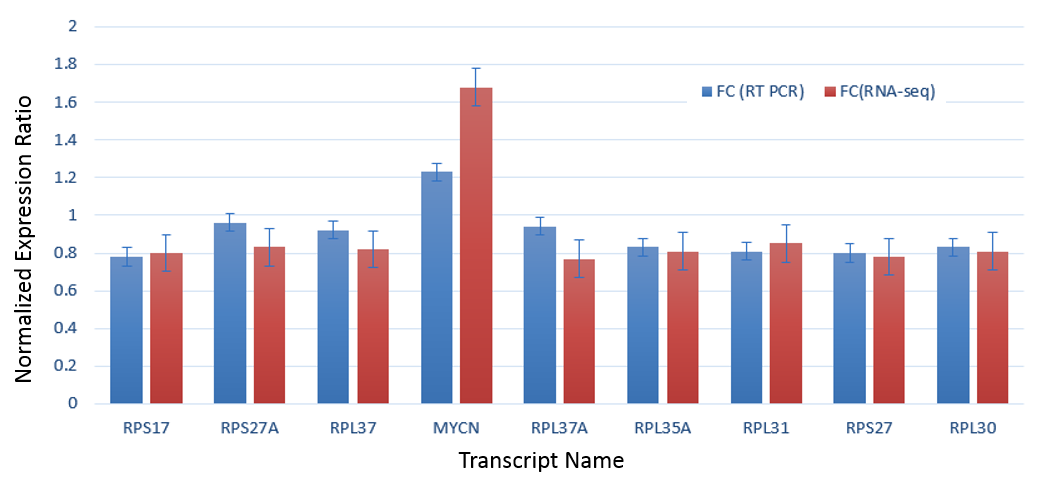


Figure S7: Scatter Plot showing the relationship between RT-PCR and RNA-seq data for the top 8 differentially expressed transcripts in MHO vs. MAO. Diagonal line represents the regression line.


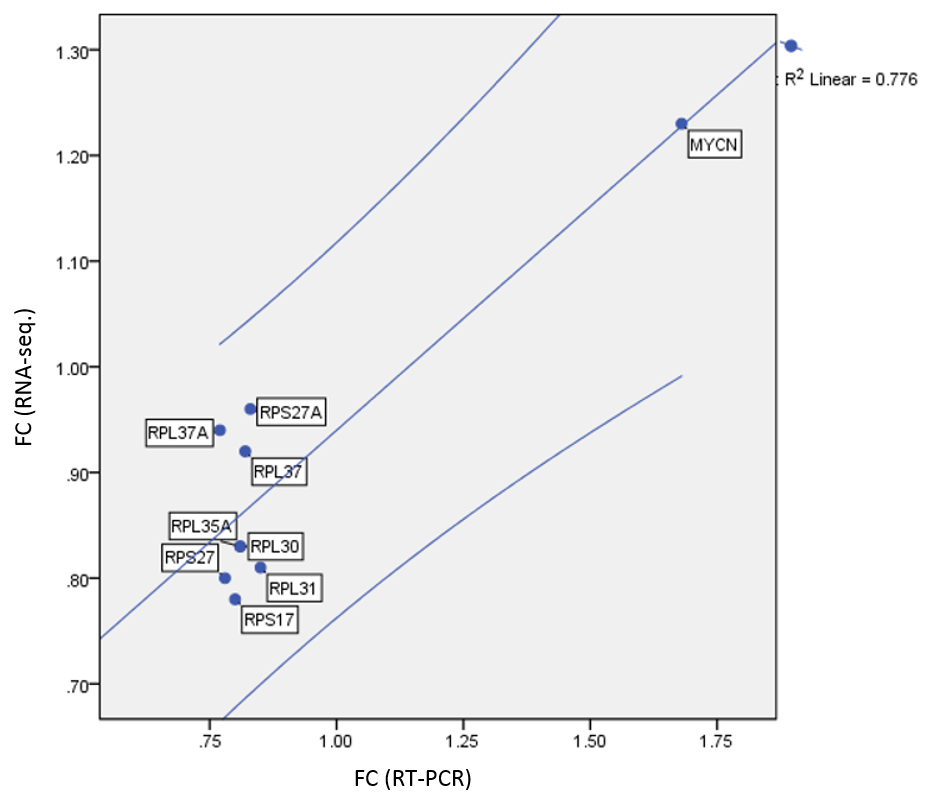


**DIFFERENTIAL EXPRESSION OF ADIPOQ GENE AND CORRELATION BETWEEN TG/HDL AND ADIPONECTIN**

Figure S8: Higher levels of (A) Total adiponectin and (B) HWM adiponectin are observed in MHO and (c) ADIPOQ gene is significantly overexpressed in the MHO group.


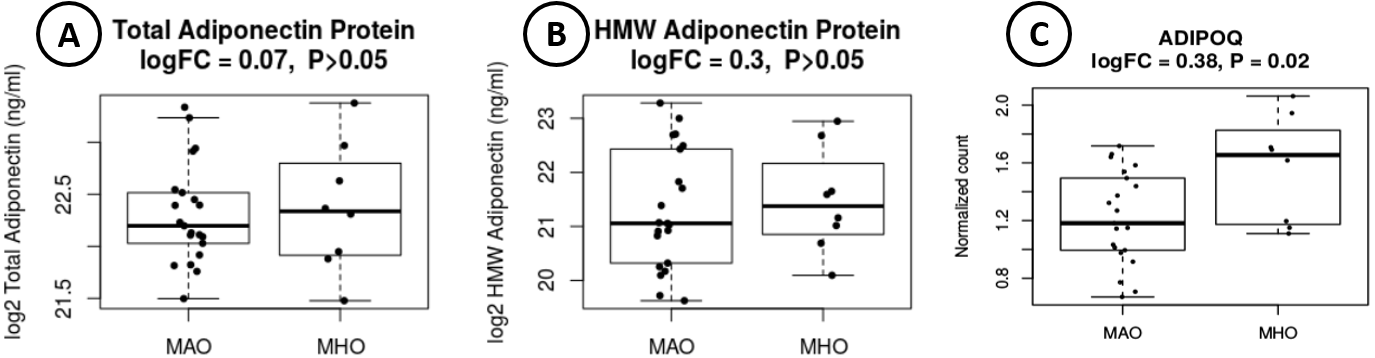


Figure S9: TG/HDL is negatively correlated with (A) total adiponectin, (B) HMW adiponectin and (C) ADIPOQ gene expression.


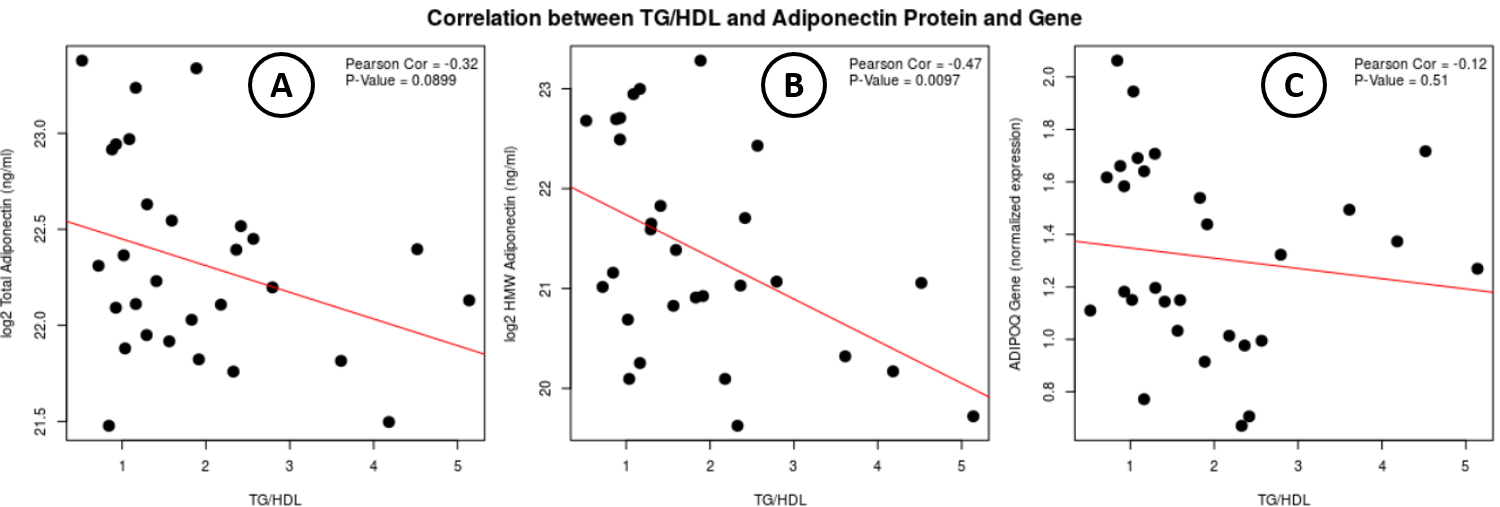


# Random Forest Analysis

**RANDOM FOREST VARIABLE SELECTION USING THE R LIBRARY VSURF**

The VSURF algorithm carries out variable selection in 3 stages as described below. The plots in Figure S10 illustrate the process, for the lightpink module.

1. Ranking of variables: 500 forests of 10,000 trees each are generated and the variables ranked by mean variable importance measure (VIM) (Figure S10A).
2. Selection of true predictors: standard deviations of VIM were used to estimate a threshold value for VIM. The threshold (dotted horizontal red line in Figure S10B) was set to the minimum prediction value given by a classification and regression tree (CART) model fitting the standard deviation curve (the minimum of the green piece-wise function in Figure S10B). For the lightpink module 10 genes with an averaged VIM > threshold (represented by the red line in Figure S10A) were selected.
3. Model performance of the true predictors measured as OOB error rate: Figure S10C indicates that the lowest OOB error (0.14) and the highest AUC (0.94) were achieved with the first 2 (RPS25 and RPS17L) of 10 genes selected in the previous step; however further checks are required to assess the stability of the model with those predictors. The 10 genes selected in the previous step were the ‘true’ important variables. They collectively predict MHO status with an AUC=0.8 (OOB error = 0.24).

Figure S10: The 3 main steps of VSURF variable selection shown graphically: (A) ranking of variables by mean VIM, (B) standard deviation of VIM for each variable and (C) OOB error for nested models fitted across the 10 true predictors of the lightpink module.


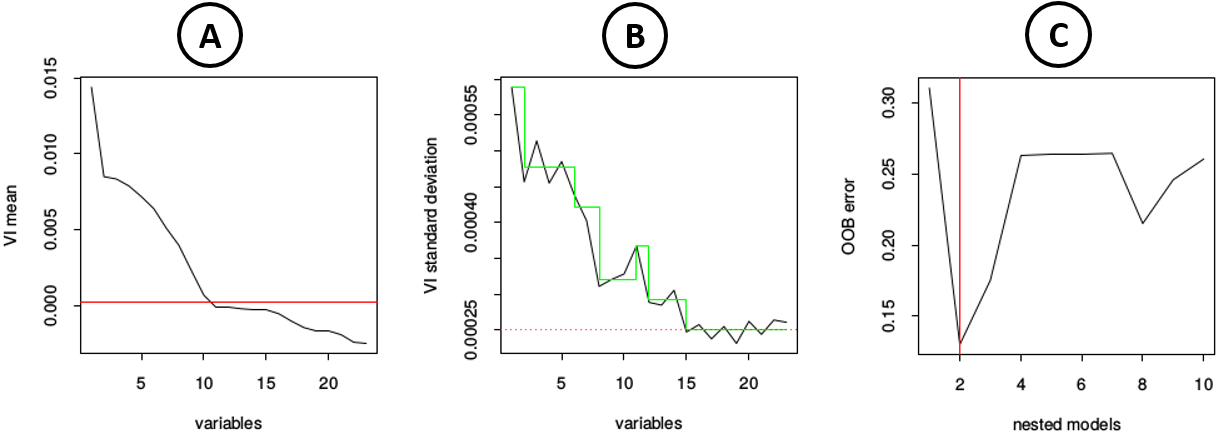


**PREDICTING LIGHTPINK MODULE GENES EXPRESSION USING INDIVIDUAL COMPONENTS OF MHO**

To identify the main metabolic component driving the relationship between the lightpink module and MHO status, we ran a Random Forest regression. The results reported in Table S3 suggest that the lipid component (TG/HDL) is by far the best predictor with a larger percentage increase in mean squared error (% IncMSE) of the genes in the lightpink module, except for ZNHIT3 and SNHG8 for which BMI is the top predictor.

For Random Forest regression, % IncMSE is a more robust and informative measure of variable importance. It is the increase in mean squared error of predictions (estimated from the out-of-bag) when a predictor variable (here each MHO component) is permuted 1000 times. The principle is identical to the one described in the methods section of the manuscript.

Table S3: Predictive power of the MHO components measured as % IncMSE for each gene in the lightpink module.

| **Lightpink Module Gene** | **BMI** | **Glucose** | **CRP** | **HOMA-IR** | **TG/HDL** |
| --- | --- | --- | --- | --- | --- |
| RPS27 | 3641.46 | -147.44 | -46.15 | -474.04 | 11083.57 |
| RPL41 | 2244.30 | 201.49 | -250.32 | -218.55 | 2554.57 |
| RPS17L | 188.04 | -98.43 | 260.54 | -113.43 | 1759.62 |
| RPL30 | 5.77 | -41.11 | 455.00 | -403.38 | 1645.68 |
| RPL37 | 186.12 | -165.22 | 59.53 | -93.02 | 1543.70 |
| RPS25 | 130.05 | -322.43 | -363.02 | -281.97 | 968.14 |
| RPL31 | 371.75 | -125.42 | 111.26 | -142.51 | 766.53 |
| RPL9 | 1042.10 | 1049.61 | 523.25 | -131.47 | 675.13 |
| RPS27A | 378.29 | 224.08 | 258.58 | -235.23 | 551.81 |
| RPL34 | 109.14 | 207.17 | -150.75 | -75.06 | 526.19 |
| RPL35A | 91.09 | -1.53 | 179.89 | -15.43 | 455.57 |
| RPL26 | 213.54 | 72.57 | 66.27 | -215.56 | 427.76 |
| RPS29 | 231.72 | 164.03 | 153.70 | 213.09 | 290.34 |
| RPL36A | 60.22 | -87.30 | -11.04 | -167.20 | 270.52 |
| RPL38 | 65.47 | -23.55 | -46.25 | -54.94 | 222.97 |
| RPS24 | 242.87 | -137.54 | -176.66 | -198.28 | 219.44 |
| RPL24 | 49.57 | 20.37 | 3.34 | -40.68 | 107.67 |
| RPS13 | 56.69 | -0.85 | 36.16 | -28.51 | 107.00 |
| COMMD6 | 0.51 | 0.77 | 0.20 | -0.86 | 2.58 |
| SNHG6 | 0.86 | 1.58 | 0.24 | -0.40 | 2.49 |
| LSM5 | 0.41 | 0.27 | 0.05 | 0.05 | 0.87 |
| ZNHIT3 | 0.23 | 0.01 | -0.08 | -0.11 | -0.07 |
| SNHG8 | 0.43 | -0.91 | -1.68 | -1.68 | -0.90 |

**TG/HDL prediction of MHO compared with the other components of MHO definition**

Random forest analyses of MHO using all MHO definition components together (Figure S11) and each separately (Figure S12) show TG/HDL ratio as the top predictor. Together, all MHO definition components predict MHO with AUC=0.85 (Figure S11) whilst TG/HDL ratio alone predicts MHO with AUC=0.93 (Figure S12).

Figure S11: Prediction of MHO using all components of MHO (definition 3).


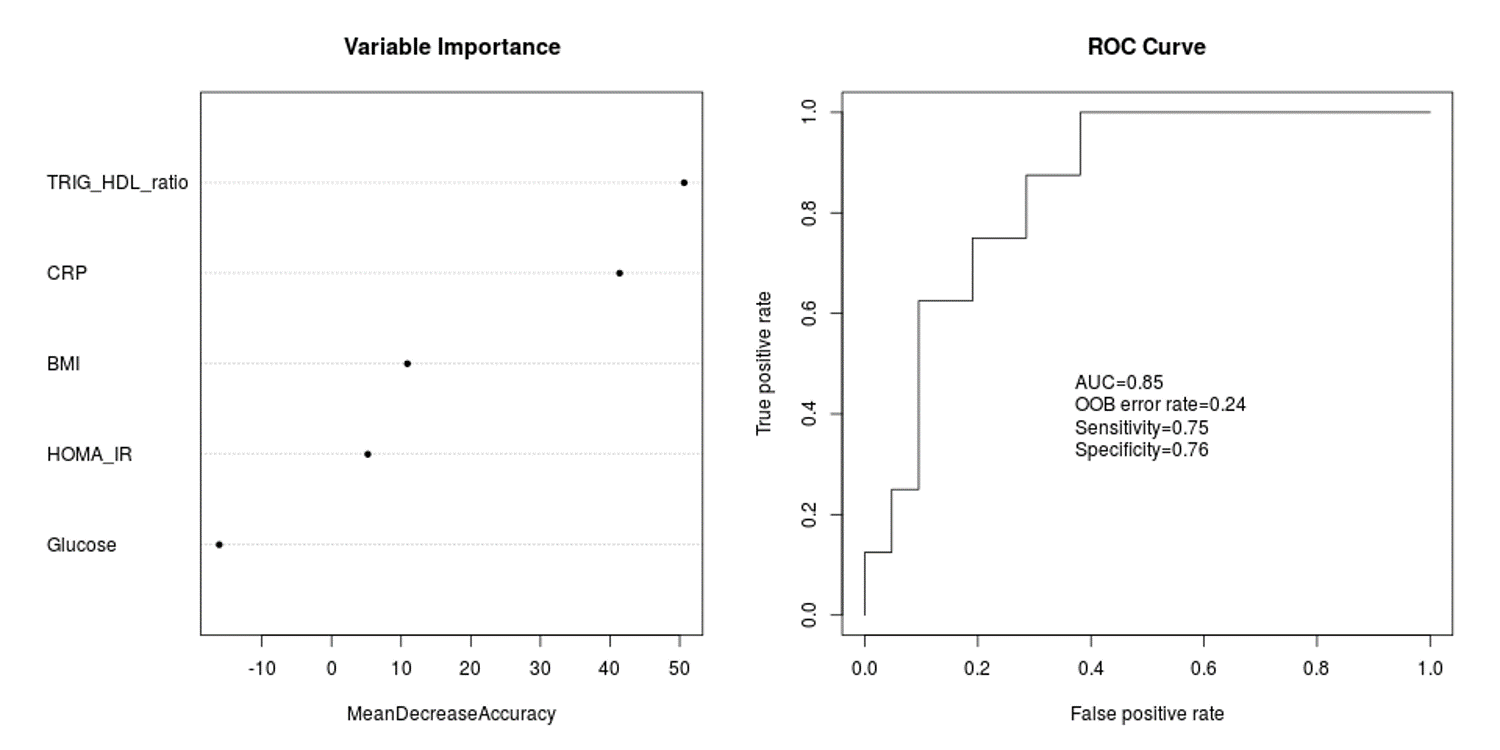


Figure S12: Prediction performance achieved with each of the MH0 components (definition 3).


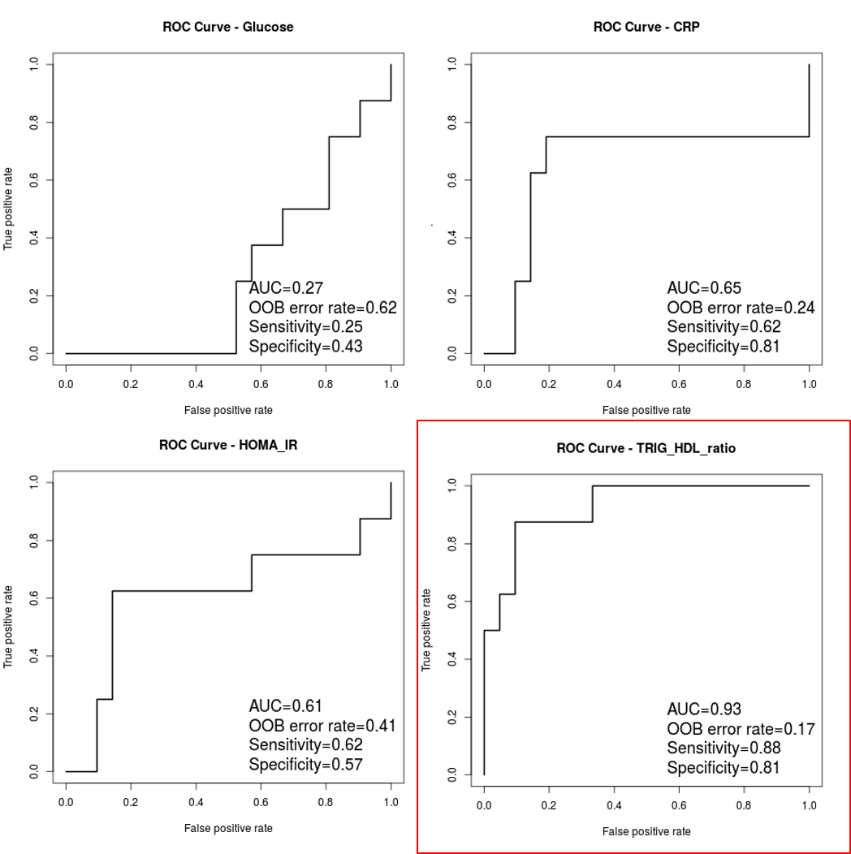


1. **MH-GRID INCLUSION AND EXCLUSION CRITERIA**

**INCLUSION CRITERIA**

- - Self-identified African-American males or females between the ages of 30 and 55 years.
  - Severe-Controlled Hypertension (SCH): SBP ≤ 140 and/or DBP ≤ 90 mmHg on a stable regimen (≥6 months) with ≥ 2 anti-hypertensive drugs (must include a diuretic).
  - Severe-Resistant Hypertension (SRH): SBP > 140 and/or DBP > 90 mmHg on a stable regimen (≥3 months) of ≥ 3 drugs (must include a diuretic).

**Controls:** Individuals with optimal blood pressure: ≤ 120/80 mmHg and normal kidney function (eGFR > 90 ml/min).

**EXCLUSION CRITERIA**

- Failure to meet the inclusion criteria.
- Secondary forms of hypertension.
- Primary chronic kidney disease or proteinuria unrelated to hypertension.
- Chronic diseases that may secondarily compromise renal function such as diabetes, chronic congestive heart failure, HIV or liver disease.
- Patients with recent hospitalizations (< 3 months).
- Unable to give informed consent.
- Pregnant or lactating women.

1. **RNA EXTRACTION AND QUANTIFICATION**

**mRNA ISOLATION**

Total RNA extraction was carried out using the MagMAX™ for Stabilized Blood Tubes RNA Isolation Kit (Life Technologies, Carlsbad, CA) following manufacturer’s recommendation. Briefly, Tempus-stabilized blood (~9 mL) was diluted with PBS and centrifuged at 5000 × g for 15 minutes at 4 °C to pellet the crude RNA. The RNA pellet was digested with Tempus proteinase and Turbo DNase, and captured using RNA binding beads and a magnetic stand. The beads were washed with wash buffers and RNA eluted in 20 uL elution buffer. To assess RNA yield and quality, a NanoDrop Spectrophotometer (Thermo Scientific, Wilmington, DE) was used to measure RNA concentration in 260/230 ratios. Additionally, an aliquot of the RNA was analyzed on an Agilent 2200 TapeStation (Agilent Technologies, Santa Clara, CA) for RINe calculation using the RNA ScreenTapes.

**mRNA LIBRARY PREPARATION**

The data used in this analysis consist of whole transcriptome mRNA sequencing data of samples from the MH-GRID study; mRNA was converted into cDNA libraries using the Illumina TruSeq Stranded Total RNA sample preparation kit (Illumina # RS-122-2303). Total RNA sample concentrations were normalized, and ribosomal RNA (rRNA) removed using biotinylated probes that selectively bind rRNA species. The resulting rRNA-depleted mRNA was fragmented using heat in the presence of divalent cations, with fragmentation times varying based on input mRNA degradation. Fragmented mRNA was converted into double-stranded cDNA, with dUTP utilized in place of dTTP in the second strand master mix. The resulting molecules were amplified via polymerase chain reaction (PCR). Final libraries were quantified by qPCR (KAPA Library Quant Kit, KAPA Biosystems # KK4824), normalized and pooled. Pooled libraries were bound to the surface of a flow cell and each bound template molecule was clonally amplified up to 1000-fold to create individual clusters. Four fluorescently labelled nucleotides were then flowed over the surface of the flow cell and incorporated into each nucleic acid chain. Fluorescence was measured for each cluster during each cycle to identify the base that was added to each cluster.

**mRNA EXPRESSION QUANTIFICATION**

Quantification of mRNA gene-level and isoform-level expression values were done in 3 steps:

(1) Adapter trimming was conducted with FastqMcf to remove remnants of sequencing primers/adapters and low-quality regions from the raw RNA-Seq read data with improvement in alignment rates.

(2) Reads were aligned to the transcriptome, using BowTie2 and the relevant reference genome (hg38).

(3) Finally, the expression level was measured using RNA-Seq by Expectation Maximization (RSEM).
